# A Clean and Tunable Mussel-Inspired Coating Technology by Enzymatic Deposition of Pseudo-Polydopamine (ψ-PDA) Thin Films from Tyramine

**DOI:** 10.3390/ijms21144873

**Published:** 2020-07-10

**Authors:** Maria Laura Alfieri, Lucia Panzella, Youri Arntz, Alessandra Napolitano, Vincent Ball, Marco d’Ischia

**Affiliations:** 1 Department of Chemical Sciences, University of Naples Federico II, Via Cintia 21, 80126 Naples, Italy; marialaura.alfieri@unina.it (M.L.A.); panzella@unina.it (L.P.); alesnapo@unina.it (A.N.); 2 Faculté de Chirurgie dentaire, Université de Strasbourg, 8 rue Sainte Elisabeth, 67000 Strasbourg, France; youri.arntz@unistra.fr; 3 Institut National de la Santé et de la Recherche Médicale, Unité Mixte de Recherche 1121, 11 rue Humann, 67085 Strasbourg, CEDEX, France

**Keywords:** polydopamine, tyramine, tyrosinase-catalyzed oxidation, low-waste coating technology, atomic force microscopy, MALDI-MS, water contact angle, cyclic voltammetry

## Abstract

The tyrosinase-catalyzed oxidation of tyramine, leading to the deposition of pseudo-polydopamine (ψ-PDA) thin films, is disclosed herein as a superior technology for surface functionalization and coating at a neutral pH and at a low substrate concentration, compared to the standard autoxidative PDA coating protocols. Smooth ψ-PDA thin films of variable thickness up to 87 nm were obtained from 1 mM tyramine by varying tyrosinase concentrations (5–100 U/mL). Compared to the PDA films obtained by the similar enzymatic oxidation of 1 mM dopamine with tyrosinase (T-PDA), ψ-PDA displayed slower deposition kinetics, lower water contact angles in the range of 11°–28°, denoting higher hydrophilicity but similar UV-vis absorption profiles, as well as electrochemical properties and antioxidant activity. MALDI-MS analysis indicated for ψ-PDA a well defined pattern of peaks compatible with dopamine tetrameric structures degraded to a variable extent. The exposure to a tyramine solution of tyrosinase-loaded alginate spheres, or films deposited on glass or polyethylene, resulted in a rapid gel-confined ψ-PDA formation with no leakage or darkening of the solution, allowing the complete recovery and re-utilization of the unreacted tyramine. In contrast, an abundant PDA precipitation outside the gel was observed with dopamine under the same conditions. The ψ-PDA deposition by tyrosinase-catalyzed tyramine oxidation is thus proposed as a controllable and low-waste technology for selective surface functionalization and coating or for clean eumelanin particle production.

## 1. Introduction

The multifunctional and universal wet surface dip-coating technology with highly adhesive polydopamine (PDA) films has dominated the scene of materials science over the past decade, since the first report of the classical autoxidation protocol from 10 mM dopamine at pH 8.5. A huge and increasing record of applications of PDA has then become available, which includes drug delivery, energy storage, molecular detection, bioimaging, catalysis, and nanobiointerface, to mention only a few [1,2,3,4,5].

Given the considerably broad scope of the original formulation, several attempts have been made to improve the coating efficiency by different approaches, e.g., by the use of chemical oxidizing agents (e.g., ammonium persulfate, sodium periodate, copper sulfate), high-salt concentrations, acidic pH, UV irradiation, the use of additives like hexamethylenediamine (HMDA), and electrochemical oxidation. In all cases, however, the improvements in film properties were counterbalanced by a loss in the ease of reaction, including mildness and versatility, e.g., for application to cellular systems [6,7,8,9,10,11,12].

However, despite the unabated interest and an ever expanding use for various substrate-independent surface functionalization applications, PDA-based technologies suffer from some limitations relating to: (i) the intrinsic toxicity of dopamine; (ii) the use of an alkaline pH, not always compatible with biological systems or alkali-unstable surfaces; (iii) the need for high dopamine concentrations (10 mM), with more than 99.9% material lost in the form of black insoluble precipitate; and (iv) difficulties to control film thickness and properties due to the slow kinetics of autoxidation [13,14,15]. The efficient control of film growth has recently been achieved by using a borate buffer, forming stable complexes with the catechol moiety of dopamine [16], or by adding resorcinol, which traps dopamine quinone [17], preventing polymerization in the presence of HMDA [18].

A possible means of bypassing the limitations inherent to the autoxidation protocol relies on the use of enzymes like tyrosinase, which can be exploited to modulate catecholamine oxidation at pH values around neutrality [19]. By this approach, good quality films were obtained from a variety of substrates that were acted upon by tyrosinase, notably including monophenols like tyramine. Tyrosinase-catalyzed, material-independent coating was thus proposed as a practical and convenient means of obtaining nanometer-thick, uniform films with diverse functionalities [19,20,21,22,23]. However, despite such a promising set of observations, the great potential of mono-phenolic (i.e., non-catecholic) tyrosinase substrates for surface functionalization and coating in view of their expected stability to autoxidation did not receive further attention.

Herein, we report the tyrosinase-catalyzed polymerization of tyramine as a mild, versatile and efficient procedure for the development of adhesive PDA-type films, for which the term of pseudo-PDA, ψ-PDA, is proposed. These ψ-PDA films could be obtained at neutral pH (i.e., 6.8) and at a much lower substrate concentration (e.g., 1 mM) compared to the standard autoxidative PDA coating protocol (typically 10 mM of dopamine). The antioxidant properties of the tyramine-derived films were also investigated in comparison with those of PDA obtained under the same conditions. Finally, protocols aimed at achieving site-specific polymerization and/or film deposition are disclosed, which involved the exposure of tyramine to confined tyrosinase. Under these conditions, the uncontrolled autoxidative deposition of a black precipitate outside the target surface, that plagues conventional PDA coating technologies, was prevented with specific advantages relating to the recovery of an unreacted precursor and the lack of interfering effects with specific applications.

## 2. Results and Discussion

### 2.1. Tyrosinase-Controlled Oxidation of Tyramine: Films Deposition

**Figure 1:**
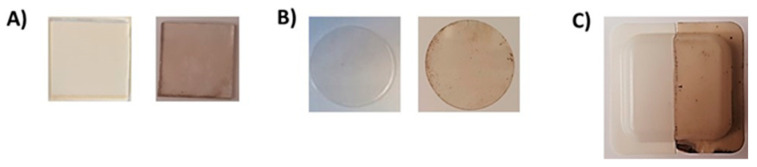
Pseudo- polydopamine (ψ-PDA) coatings on various materials: (**A**) quartz; (**B**) polycarbonate; and (**C**) polystyrene. On the left, the uncoated materials are shown.

The immersion of various substrates in a solution containing 1 mM tyramine at pH 6.8 in the presence of variable amounts of tyrosinase in the 20–100 U/mL range resulted in the deposition after 24 h of dark films resembling PDA (Figure 1).

**Figure 2:**
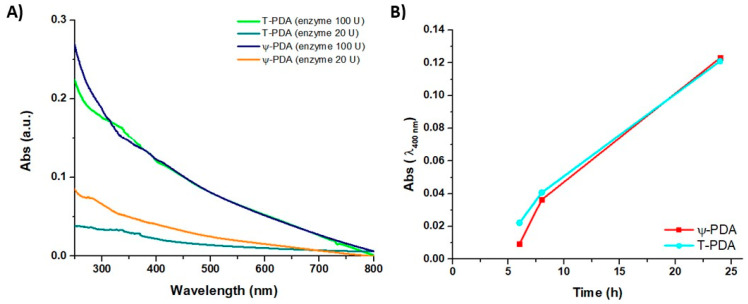
(**A**) UV-vis spectra of the films obtained from 1 mM tyramine by varying tyrosinase concentrations (20 and 100 U/mL) compared to the PDA films obtained by the similar enzymatic oxidation of 1 mM dopamine with tyrosinase (tyrosinase-PDA, T-PDA). (**B**) Evolution of the UV-vis spectra over time of the ψ-PDA or T-PDA films in the presence of tyrosinase (100 U/mL) at a selected wavelength (400 nm).

Figure 2A shows the absorption spectra of the films produced from 1 mM tyramine and variable amounts of tyrosinase (20 and 100 U/mL) after 24 h against the films produced by the oxidation of 1 mM dopamine under the same conditions (tyrosinase-PDA, T-PDA). The data showed similar UV-vis profiles for the two amines and indicated significant deposition of PDA films from 1 mM catecholamine concentration. It is noted that in the autoxidation experiments at least 10 mM dopamine is required for film deposition. The evolution of the film-forming properties of ψ-PDA or T-PDA, shown in Figure 2B, was also investigated by dipping quartz substrates in 1 mM tyramine or dopamine solution undergoing oxidation in the presence of tyrosinase (100 U/mL) in carbonate buffer (pH 6.8) at different times (6, 8 and 24 h) after oxidation had started. The UV-vis analysis showed that the absorbance of either the ψ-PDA and T-PDA films is alike over time with the highest extent of coating after 24 h.

The tyrosinase-catalyzed oxidation kinetics of 1 mM tyramine in carbonate buffer pH 6.8 were also monitored by UV-vis over 24 h compared to the enzymatic oxidation of 1 mM dopamine with tyrosinase (20 U/mL) (Figure S1). After a few hours a progressive consumption of the starting material was observed, faster in the case of dopamine, together with the usual color change (from colorless to black) and the precipitation of black melanin-like materials after 24 h.

### 2.2. Kinetic of Films Deposition: Quartz Crystal Microbalance Methodology

**Figure 3:**
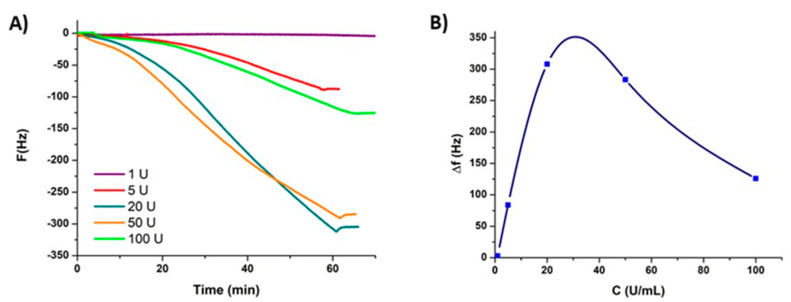
(**A**) Quartz crystal microbalance methodology (QCM-D) experiment with frequency variations at the third overtone as a function of time during the ψ-PDA deposition in the presence of tyrosinase at different concentrations up to one hour. (**B**) Evolution of the frequency as a function of the tyrosinase concentration after 1 h of deposition.

The deposition kinetics of the film from tyramine in the presence of variable amounts of tyrosinase (1–100 U/mL) were then followed in situ using the quartz crystal microbalance methodology (QCM-D). After an equilibration time of ca. 5 min with a carbonate buffer solution (*∆f/ν* < 1 Hz) the tyramine solutions were introduced in the cell and let it flow for about 1 h. As shown in Figure 3, the deposition kinetics of ψ-PDA are much faster in the presence of 20 or 50 U/mL of enzyme than with 100 U/mL of tyrosinase, the latter appearing rather smooth over the observed period of time.

For comparative purposes, the same technique and procedure were applied to the dopamine enzymatic oxidation mixture, performed under the same conditions adopted in the case of tyramine and in the presence of 20 U/mL of enzyme. The data reported in Figure S2 show that after 40 min the deposition of T-PDA is more significant than that of ψ-PDA. This observation is in agreement with the notoriously faster reactions of tyrosinase with catechols with respect to monophenols, which often display an induction time [24,25].

Increasing the amount of tyrosinase resulted in a maximal deposition rate followed by a drop in the reaction kinetics after the initial rise. This may well be attributed to a progressive inactivation of the enzyme when its concentration and hence the rate of tyrosine oxidation increases, leading to faster deposition kinetics followed by a progressive coating of the enzyme by ψ-PDA at a higher enzyme concentration. This capping of the enzyme by ψ-PDA may lead to its inactivation.

The thickness of the films, reported in Table 1, was calculated, based on the observation that the reduced frequency changes (*Δf/ν*) overlap and the dissipation remains small, using the Sauerbrey equation [26] from the QCM-D data as expressed by (1):

(1) $$\Delta m=-C*\frac{1}{v}*\Delta f$$

where *ν* is the overtone number, *C* is a constant that depends on the property of the crystal used, *∆m* is the mass deposited at the quartz surface, and *∆f* is the variation in the resonant frequency induced by the deposited mass.

### 2.3. Morphological Characterization

**Figure 4:**
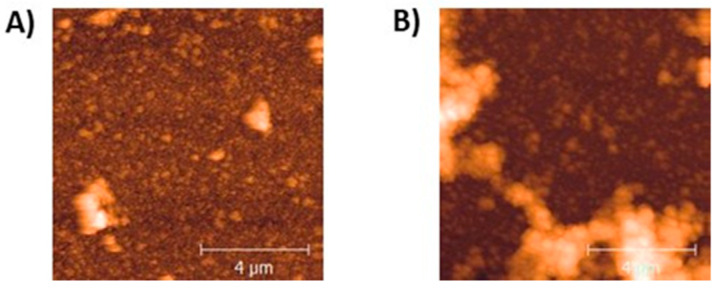
Atomic force microscopy (AFM) image of a representative region of the ψ-PDA film sample, after 1 h of deposition onto the quartz crystal sensor, in the presence of 20 U/mL tyrosinase (**A**) or (**B**) a T-PDA film sample in the presence of 20 U/mL tyrosinase. Roughness: 45.5 nm (**A**), 113.5 nm (**B**). Film thickness: 87 ± 7 nm (**A**), 25.3 ± 8 nm (**B**).

In a subsequent series of experiments, the morphology and surface properties of the ψ-PDA films were investigated. Atomic force microscopy (AFM) images reported in Figure 4A and Figure S3 indicated smooth films with a thickness varying with tyrosinase levels within the upper limit of 87 nm (Table 2). Compared to the ψ-PDA film, T-PDA film (Figure 4B), obtained under the same enzymatic oxidation conditions, displayed a higher average roughness.

Notably, these data confirm the trendline observed in Table 1 although the experimental thickness values could be different from those calculated using the Sauerbrey equation.

The water contact angle (WCA) measurements indicated relatively hydrophilic films not exceeding 28°, to be compared with a value of 32° for the T-PDA produced under the same conditions (in the presence of 20 U/mL of tyrosinase) (Table 2).

Whereas the maximal film deposition was observed with an enzyme concentration around 20 U/mL (Figure 3), the size of the colloids obtained in the solution was found to increase monotonically up to 100 U/mL in enzyme as shown in Table 3. This difference can be attributed to the different kinetics and mechanisms of film deposition and particle aggregation, with the former depending on the critical levels of adhesive species and adsorption processes which do not affect large particle growth.

### 2.4. Electrochemical Properties

**Figure 5:**
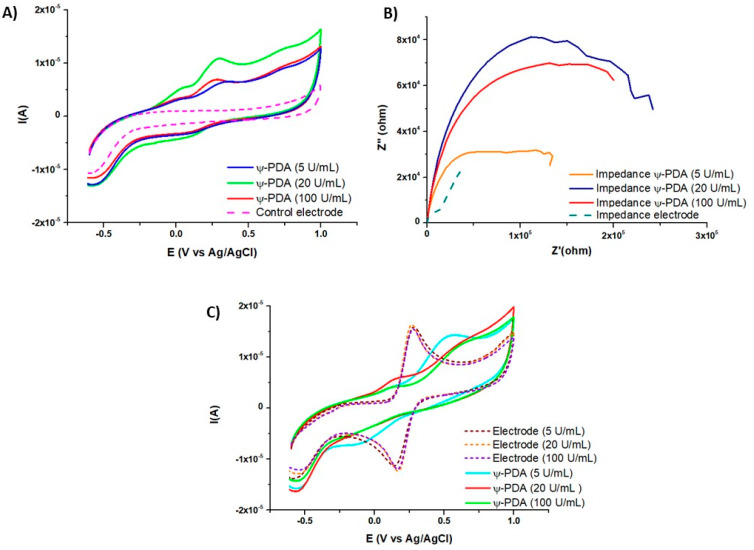
Electrochemical analysis of the ψ-PDA films deposited on amorphous carbon-working electrodes as a function of the enzyme concentration used to produce them. (**A**) Capacitive curves of the films in the presence of a carbonate buffer at pH 6.8 after 1 h of electrode deposition in the tyramine/enzyme mixtures. (**B**) Electrochemical impedance spectra, in the Nyquist representation and in the presence of the redox probe in the solution of the ψ-PDA films as a function of the enzyme concentration used to produce them. (**C**) Comparison of the cyclic voltammetry (CV) curves of potassium hexacyanoferrate as a redox probe on the pristine electrodes (dashed lines) and the same electrodes covered with the ψ-PDA films.

Subsequent experiments were directed to assess the redox properties of the tyramine-derived films by the means of cyclic voltammetry (CV). The data in Figure 5 indicate a significant response for the tyramine film obtained with 20 U/mL of enzyme after 1 h deposition compared to the ones obtained with 5 or 100 U/mL. In agreement with the AFM and QCM-D data, the enzyme concentration of 20 U/mL appears optimal for the film deposition. Indeed, the area under the capacitive CV curves, performed in the absence of a soluble electrochemical redox probe and corresponding to the film capacitance, is the highest for the film deposited in the presence of this enzyme concentration (Figure 5A). This trend is confirmed by electrochemical impedance spectroscopy, where the modulus of the films’ impedance (Z’^2^ + Z″^2^) is higher in the presence of 20 U/mL of the enzyme (Figure 5B). In addition, when considering the CV curves of potassium hexacyanoferrate in the presence of the deposited films (Figure 5C), it appears that the films deposited in the presence of 5 U/mL display some residual permeability of the redox probe to the electrode, whereas those obtained at higher enzyme concentrations are impermeable, indicating the formation of conformal films in those cases.

**Figure 6:**
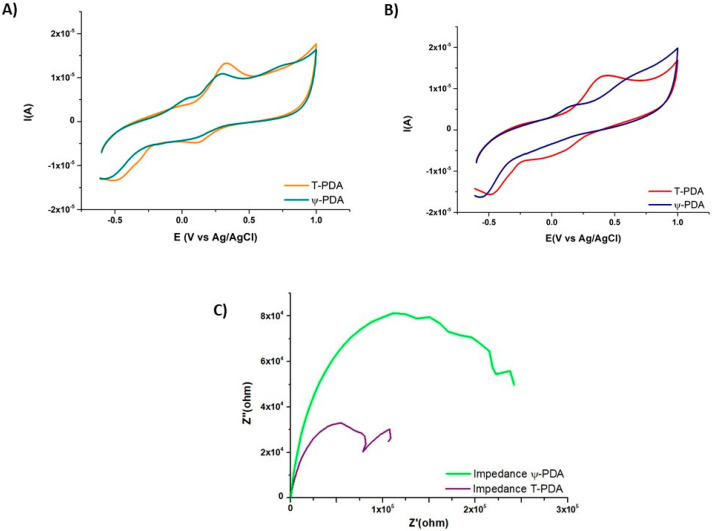
Comparative electrochemical analysis of the ψ-PDA and of the T-PDA films deposited during 1 h on carbon working electrodes in the presence of 20 U/mL of tyrosinase. (**A**) CV curves obtained after 1 h film deposition in the carbonate buffer at pH 6.8. (**B**) CV curves obtained after the film deposition and in the presence of potassium hexacyanoferrate. (**C**) Electrochemical impedance spectra of the ψ-PDA and T-PDA films in the Nyquist representation and in the presence of the redox probe.

The comparison of the electrochemical behavior of the ψ-PDA and T-PDA films, obtained in the presence of 20 U/mL, shows that both are electroactive and leading to an irreversible oxidation-reduction wave in the absence of the external redox probe (Figure 6A). When both deposits are exposed to potassium hexacyanoferrate, it appears that the ψ-PDA film is more impermeable than that of the T-PDA film (Figure 6B) which is also in agreement with the marked difference in their impedance spectra (Figure 6C). From a semi-quantitative analysis of those spectra, in the low frequency domain, it appears that the ψ-PDA films display a higher resistance to the diffusion of the redox probe to the electrode than that of the T-PDA film.

### 2.5. Structural Characterization

The structural characteristics of the bulk precipitates and films of ψ-PDA and T-PDA produced by tyramine-tyrosinase and by dopamine-tyrosinase oxidation under the same conditions were compared using the matrix-assisted laser desorption/ionization-mass spectrometry (MALDI-MS). Notably, whilst the bulk precipitates displayed partially overlapping peak patterns, indicating a gross structural similarity, distinct spectral patterns were observed for the two films, with few minor peaks in common (Figure S4). The main peaks that were considered for structural analysis are reported in Table 4.

**Figure 7:**
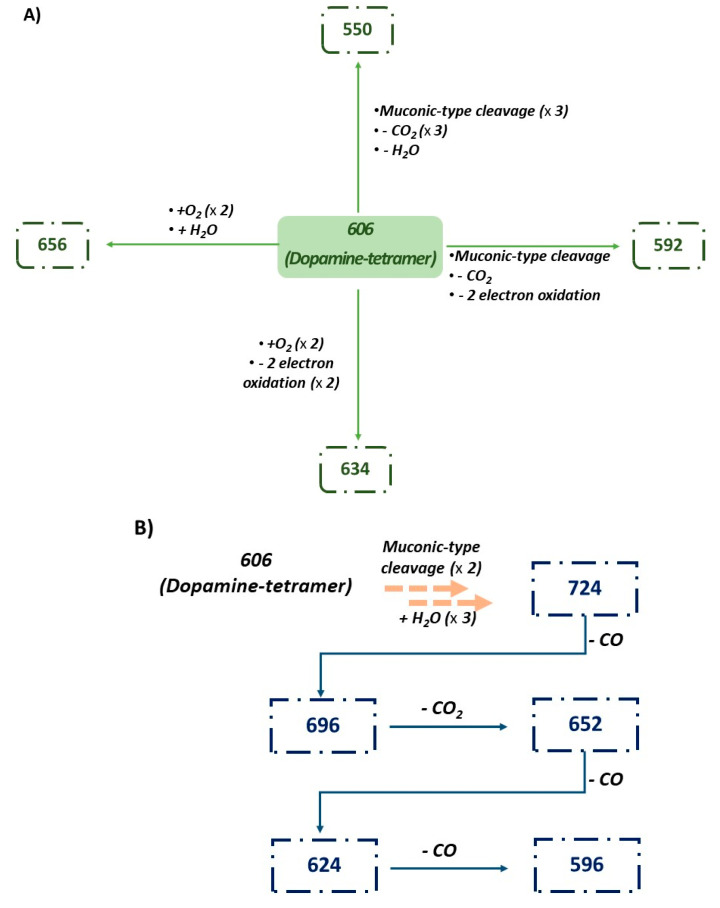
Proposed origin of the main peaks of the MALDI-MS spectra of the ψ-PDA (**A**) and the T–PDA (**B**) films.

As represented in Figure 7, the proposed structural modifications, accounting for the observed peaks, can be rationalized starting from the dopamine tetrameric structures that underwent a series of muconic-type cleavage of o-quinones with the formation of two carboxyl groups, decarboxylation and decarbonylation, electron oxidation (e.g., conversion to quinone), oxygenation (e.g., hydroxylation, quinone epoxidation) and hydration/dehydration steps. Overall, these data confirmed a PDA-like composition for the ψ-PDA films.

### 2.6. Antioxidant Activity

In a separate set of experiments, the antioxidant properties of the ψ-PDA suspensions and films were investigated in comparison with those of the T-PDA.

For the ψ-PDA bulk precipitate, an EC_50_ value of 54.0 ± 0.4 µg/mL was obtained in the 2,2-diphenyl-1-picrylhydrazyl (DPPH) assay, lower than that measured for the T-PDA polymer (91.3 ± 0.3 µg/mL) (Figure S5). However, these polymers exhibit an antioxidant power much lower than that of other phenolic polymers [27], as expected considering the extensive structural degradation, that negatively impacts on the antioxidant properties, in agreement with the MALDI-MS data.

**Figure 8:**
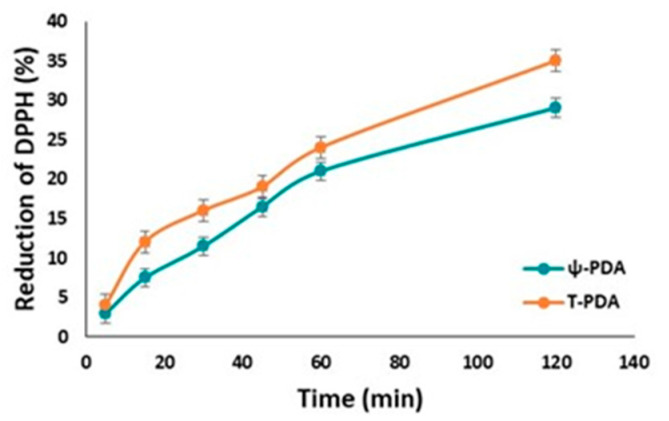
Reduction of DPPH (50 µM) over time by the immersion of the glass substrates of ψ-PDA and T-PDA.

The antioxidant properties of the films obtained by the oxidation of tyramine or dopamine in the presence of 20 U/mL of tyrosinase were then evaluated. As shown in Figure 8, the hydrogen donation capacity was well detectable, although it was not possible to assess these data on a quantitative basis and draw reliable comparative analyses with polymer suspensions. The antioxidant power of the ψ-PDA film proved to be comparable to that of the T-PDA film based on the DPPH consumption at 2 h.

**Figure 9:**
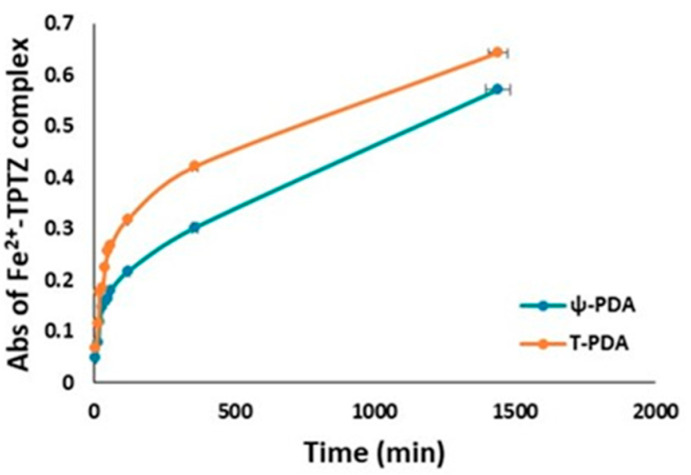
Kinetics of the development of the absorption of the Fe^2+^-tripyridyl triazine complex obtained by the immersion of glass substrates coated with ψ-PDA and T-PDA.

A similar procedure was followed to evaluate the ferric reducing antioxidant power (FRAP) of the ψ-PDA and T-PDA polymers. The results reported in Figure S6 show a reducing capacity equal to about 0.15 equivalent of Trolox in the case of the ψ-PDA polymer, higher than that obtained for the T-PDA polymer. An opposite trend was observed with the films, since those obtained by tyrosinase-tyramine exhibited an antioxidant power comparably or slightly worse than that of the tyrosinase-dopamine films (Figure 9).

### 2.7. Tyrosinase-Loaded Alginate

Since the main goal of this study was the development of a clean protocol for the generation of an adhesive functionalizing polymer with no waste of unreacted precursor or the massive precipitation of black polymer, in a final set of experiments, tyrosinase was incorporated into alginate gel, both spheres and films on glass, and the enzyme-containing gels were exposed to 1 mM tyramine, and for comparison, dopamine, in a buffer at pH 6.8.

**Figure 10:**
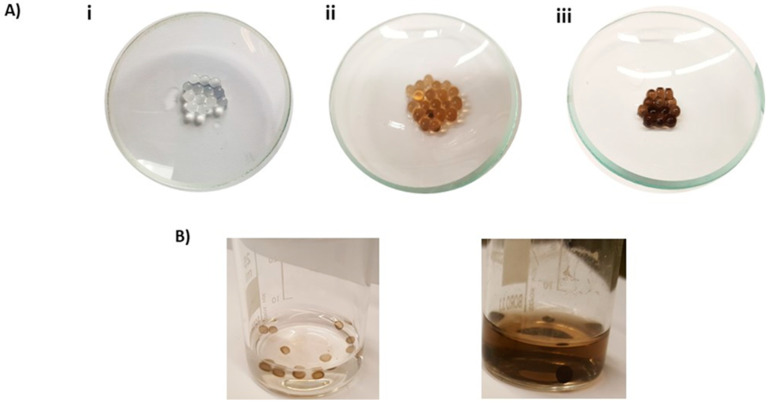
(**A**) Calcium alginate hydrogel beads (i) incorporating 0.1% (ii) or 0.5 % (iii) tyrosinase and dipped into tyramine solution; (**B**) tyrosinase (0.1%) alginate hydrogel beads dipped in 1 mM tyramine (left) or dopamine solution (right) over 2 h.

Figure 10 shows the separated gel spheres and the reaction vessel solutions for both substrates. No precipitate was apparent in the tyramine solution, despite the visible darkening of the alginate spheres, in contrast with the case of dopamine, where abundant dark precipitate was eventually produced after several hours by autoxidation even at a neutral pH. Similar results were obtained on alginate films layered on glass or other surfaces (Figure S7). After the reaction was complete, the ψ-PDA gel was removed from the solution and the tyramine-containing solution could be re-used for other reactions for a high number of repeated coating/functionalization experiments. The reported procedure allowed to produce the ψ-PDA films or polymer-functionalized gels in a facile, clean and efficient manner with considerable material saving. The enzyme appears to be irreversibly trapped in the sodium alginate gel. It maintains its activity even though its amount inside the gel may be low. The lack of spoilage of any material via leakage was demonstrated by the lack of visible tyramine polymerization outside the tyrosinase-containing gel (Figure 10A).

## 3. Materials and Methods

Tyramine, dopamine hydrochloride, tyrosinase from mushroom (2687 U/mg), potassium hexacyanoferrate, 2,5-dihydroxybenzoic acid, 2,2-diphenyl-1-picrylhydrazyl (DPPH), ferric chloride (III) hexahydrate and 2,4,6-tri(2-pyridyl)-s-triazine (TPTZ) were purchased from Sigma Aldrich (Milano, Italy) and used without further purification. The substrates (quartzes and borosilicate glass coverslips) were rinsed with a piranha mixture H_2_SO_4_/30% H_2_O_2_ 5:1 for 24 h and then extensively washed with water and dried under vacuum.

**General procedure for substrate coating**. Tyrosinase (1–100 U/mL) was added to a 1 mM solution of tyramine or dopamine in a 0.05 M sodium carbonate buffer pH = 6.8 under vigorous stirring. Substrates were dipped into the reaction mixture after the complete dissolution of the starting materials and left under stirring up to 24 h, then rinsed with distilled water, sonicated and dried under vacuum. The UV-vis spectra were recorded on a Jasco V-730 Spectrophotometer (Jasco Corporation, Lecco, Italy).

**Quartz crystal microbalance methodology**. The measurements were carried out in the electrochemical module (Q-Sense, Göteborg, Sweden) of a quartz crystal microbalance with dissipation monitoring (QCM-D). The mass change results from the variation of the resonance frequency (−*Δf*) of an oscillating quartz crystal when the material was adsorbed onto its surface from a solution and the dissipation factor (*ΔD*) provided a measure of the energy loss in the system. In particular, the changes in the oscillation frequency of the quartz crystal are followed at its third, fifth seventh and ninth overtones at 15, 25, 35 and 45 MHz, respectively. Based on the assumption that the film is homogeneous, rigid and non-slipping, the normalized frequency changes, −*Δf/ν*, is proportional to the mass deposited on the crystal per unit area accordingly to the Sauerbrey relation [26]. The gold-coated sensor crystals consist of a quartz disk and electrodes sputtered on both sides of the quartz disk, consisting of a gold surface layer with a chromium underlayer to strengthen the adhesion. The Q-Sense sensor crystals were cleaned using a Plasma Cleaner PDC-32G-2 (Harrick Plasma, Ithaca, NY, USA) for 15 min before use. The tyramine or dopamine solutions were injected into the cell during one hour at a flow rate of 0.25 μL/min using a peristaltic pump.

**Water contact angle**. A contact angle goniometer (digidrop-gbx, Bourg-de-Peage, France), equipped with video capture, was used for the water contact angle analyses. Then, 1 μL of distilled water was dropped on the air side surface of the substrate.

**AFM analysis**. The acquisition of the AFM topography was made with a commercial microscope (Bio-CATALYST, Bruker, Santa Barbara, CA, USA) by contact mode and in liquid phase. The cantilevers used were the Scan-assist Fluid (Bruker) with a nominal spring constant of 0.7 N/m and a type radius of 20 nm. The images were acquired with a resolution of 512 × 512 and a scan rate of 0.5 Hz. The film thickness was determined by measuring the difference in height between the needle scratched area and the native area of the film. The measurement was repeated three times. The roughness (Ra) was estimated on 20 µm × 20 µm, using the freeware software Gwyddion (GNU, Boston, MA, USA), dedicated to SPM microscopy analysis.

**Dynamic light scattering (DLS)**. The DLS measurements were conducted on a Nano ZS (Malvern Instruments, UK; operative scattering angle: 173° and wavelength: 632.8 nm). To a 1 mM solution of tyramine in 0.05 M sodium carbonate buffer pH = 6.8, tyrosinase (5, 20 or 100 U/mL) was added and after 1 h the size of the colloids was measured.

**Cyclic Voltammetry.** The electrochemical characterization of the films deposited from the solution was performed by means of cyclic voltammetry and electrochemical impedance spectroscopy using a three-electrode set-up (ChI 604B, CH Instruments, Houston, TX, USA). Before the deposition, the amorphous carbon working electrode (ref. CHI 104, CH Instruments, Houston, TX, USA) was cleaned with Al_2_O_3_ slurries (Escil, Villeurbanne, France) (1.0 and 0.1 µm in diameter) and then washed with water in an ultrasound bath for 20 min. To test the surface state of the working electrode, a cyclic voltammetry scan between −0.6 and +1.0 V vs. the reference electrode, in the presence of 1 mM potassium hexacyanoferrate, was performed at a potential sweep rate of 100 mV·s^−1^. The electrode was used for film deposition only if the oxidation and reduction peak potentials were separated by less than 80 mV (the theoretical peak separation for a one-electron process being 59 mV at 25 °C) [28], otherwise the electrode was cleaned again. After the film deposition for one hour, the capacitive CV curve of the coatings were acquired in the presence of a buffer at a scan rate of 100 mV·s^−1^. After that, the pure buffer was replaced by a 1 mM potassium hexacyanoferrate-containing solution to investigate the film permeability to this redox probe, at the same potential scan rate. Finally, the impedance spectra were acquired in the presence of the redox probe at a constant potential (vs. the reference electrode) corresponding to the oxidation peak potential measured on the pristine electrode with a potential modulation of ±5 mV with a frequency changing from 10^5^ to 10^−2^ Hz.

**MALDI-MS analysis**. The positive reflectron MALDI spectra were recorded on a AB Sciex TOF/TOF 5800 instrument using 2,5-dihydroxybenzoic acid as the matrix. The pigments were applied to the MALDI target plate from a fine suspension in methanol, obtained by homogenization with a glass to glass potter or scratched directly from the substrate surface, and air-dried for analysis. The displayed spectra data were acquired over a mass range of *m/z* 100–4000 Da, and each mass spectrum was collected from the accumulation of 15.000 laser shots. The Data Explorer software, provided by the manufacturers, was used to analyze the raw data reported as monoisotopic masses.

**The 2,2-diphenyl-1-picrylhydrazyl (DPPH) assay**. The assay was performed as described [29]. To a 200 μM DPPH solution in methanol, a proper amount of a 2 mg/mL methanol suspension of each sample (homogenized with a glass/glass potter) was added. The mixtures were taken under stirring at room temperature and after 15 min the absorbance at 515 nm was determined. The experiments were run in triplicate. The glass substrates, coated as described above, were instead immersed in a 50 μM solution of DPPH (15 mL) for 2 h and the antioxidant power was evaluated by recording the UV-vis spectra every 5 min. The experiment was repeated on different substrates and showed an acceptable reproducibility.

**Ferric reducing/antioxidant power (FRAP) assay**. The assay was performed as described [30]. To 3.6 mL of a freshly prepared solution of 20 mM FeCl_3_ and 10 mM 2,4,6-tris(2-pirydyl)-s-triazine in 0.3 M acetate buffer (pH 3.6) (FRAP reagent), a proper amount of a fine methanol suspension (2 mg/mL) of each sample was added, and the mixtures were taken under vigorous stirring at room temperature. The reduction of Fe^3+^ to Fe^2+^ was monitored by measuring the absorbance at 593 nm after 10 min. The results were expressed as Trolox equivalents. The experiments were run in triplicate. The glass substrates were instead immersed for 24 h in 15 mL of the FRAP reagent.

**Sodium alginate hydrogel preparation**. For the preparation of alginate hydrogels, sodium alginate was dissolved in distilled water (20 mL, 2% *w*/*w*) and then tyrosinase at 0.1 or 0.5% in water was added. The thus prepared gel was dropped using a syringe in a 0.1 M solution of CaCl_2_ allowing the alginate reticulation for few minutes or spread on a smooth surface followed by dipping in the CaCl_2_ solution for about 30 s. The beads and films thus obtained were washed in distilled water and dipped in tyramine solution (1 mM) for 2 h.

## 4. Conclusions

Produced by the tyrosinase-catalyzed polymerization of tyramine, ψ-PDA is disclosed as a valuable alternative to the classical PDA-coating technology with considerable advantages that relate to the enzymatic control of film deposition. The films of ψ-PDA display structural and physicochemical properties similar to those of T-PDA and similar antioxidant activity. The confinement or immobilization of tyrosinase at the desired site of functionalization resulted moreover in a highly clean film deposition procedure with the complete recovery and multiple reuse of unreacted tyramine and no unwanted substrate-consuming autoxidation processes, which may decrease its cost effectiveness and may interfere with the film deposition procedure.

## Figures and Tables

**Table 1:** Thickness values for the pseudo- polydopamine (ψ-PDA) and tyrosinase-PDA (T-PDA) films calculated by the Sauerbrey equation.

ψ-PDA Films	1 U	5 U	20 U	50 U	100 U	T-PDA Film	20 U
**Thickness (nm)**	3.5	11.9	42.4	39.5	15.7		36.9

**Table 2:** Characterization of the thin films from the ψ-PDA and T-PDA on crystal quartz.

Film	Thickness (nm)	Roughness (nm)	Water Contact Angle (deg)
**ψ-PDA** 100 U/mL	58 ± 4	12.8	22.3
**ψ-PDA** 50 U/mL	63.3 ± 5	28.7	17.1
**ψ-PDA** 20 U/mL	87 ± 7	45.5	28.6
**T-PDA** 20 U/mL	25.3 ± 8	113.5	32.1

**Table 3:** Dynamic light scattering (DLS) of the tyramine solutions in the presence of 5, 20 or 100 U/mL of tyrosinase, in the order after 1 h.

	5 U	20 U	100 U
**DLS (nm)**	57.45 ± 18	129.4 ± 28	149.3 ± 52

**Table 4:** Main peaks considered for structural analysis.

ψ-PDA film *Benchmark: Tyramine (137 g/mol)*		T-PDA film *Benchmark: Dopamine (153 g/mol)*	
Observed Peaks (Clusters)	MW of Molecular Species	Observed Peaks (Clusters)	MW of Molecular Species
573 (Na^+^), 589 (K^+^)	550		
615 (Na^+^), 631 (K^+^)	592		
		619 (Na^+^), 635 (K^+^)	596
		625 (H^+^), 647 (Na^+^), 663 (K^+^)	624
637(Na^+^), 653 (K^+^)	614		
		653 (H^+^), 675 (Na^+^), 691 (K^+^)	652
657(Na^+^), 673 (K^+^)	634		
679 (Na^+^), 695 (K^+^)	656		
		719 (Na^+^), 735 (K^+^)	696
		741 (Na^+^), 757 (K^+^)	718
		747 (Na^+^), 763 (K^+^)	724
		769 (Na^+^), 785 (K^+^)	746

## Supplementary Materials

Supplementary materials can be found at https://www.mdpi.com/1422-0067/21/14/4873/s1.

## Abbreviations

PDA	Polydopamine
MALDI-MS	Matrix-assisted laser desorption/ionization–mass spectrometry
DPPH	2,2-diphenyl-1-picrylhydrazyl
TPTZ	2,4,6-tri(2-pyridyl)-s-triazine
AFM	Atomic force microscopy
DLS	Dynamic light scattering
WCA	Water contact angle
CV	Cyclic voltammetry
