# Supplementary material for: A Clean and Tunable Mussel-Inspired Coating Technology by Enzymatic Deposition of Pseudo-Polydopamine (ψ-PDA) Thin Films from Tyramine
Source: Int J Mol Sci. 2020 Jul 10;21(14):4873. doi: 10.3390/ijms21144873 (PMC7402308; doi:10.3390/ijms21144873)
Supplement: Supplementary file 1 [file ijms-21-04873-s001.pdf]

Supporting Information

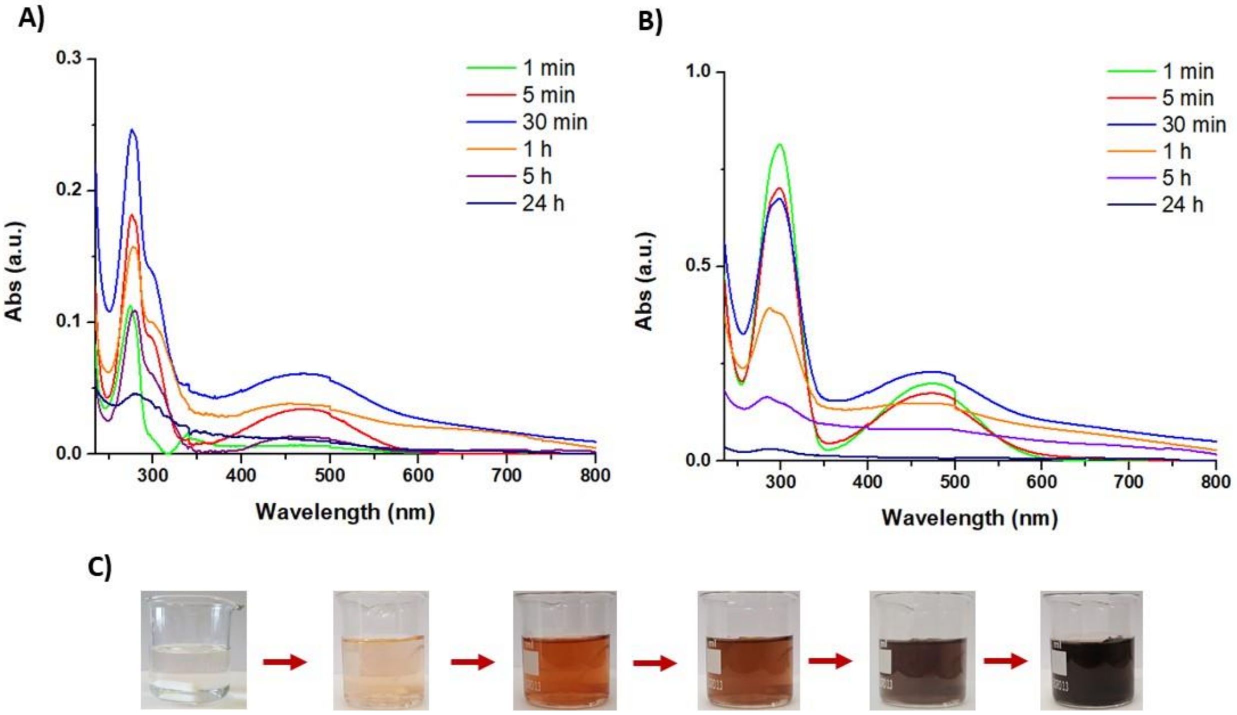

Figure S1. Evolution of the UV-vis absorption spectra of tyramine solutions (1 mM) (**A**) and dopamine solutions (**B**) during the enzymatic reaction in carbonate buffer at pH 6.8 over 24 h (diluted 10-fold before the measurement of the spectrum). (**C**) Digital pictures of catecholamine oxidation in the presence of 20 U/mL of tyrosinase.

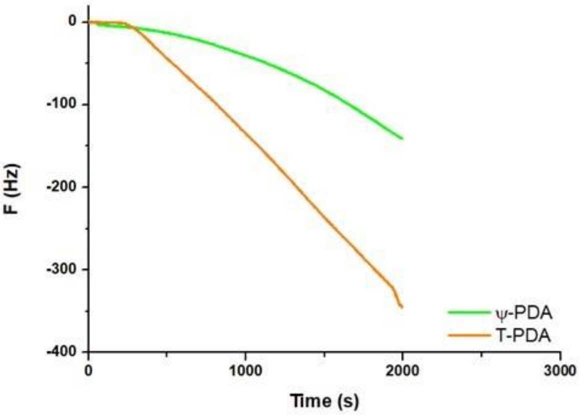

Figure S2. Kinetics of film deposition of tyramine or dopamine in the presence of 20 U/mL of tyrosinase.

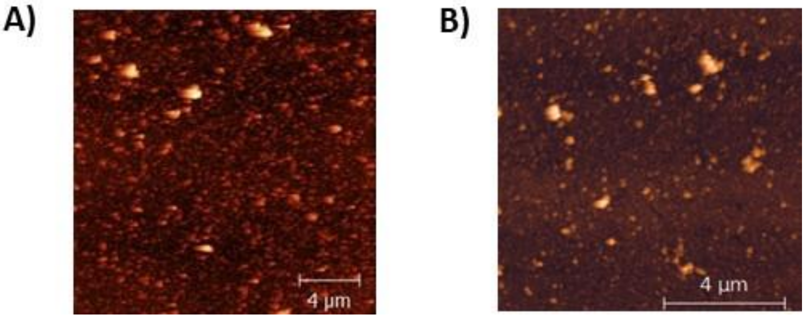

Figure S3. AFM image of a representative region of the ψ-PDA film sample, after 1 h of deposition onto the quartz crystal sensor, in the presence of 50 U/mL (**A**) and 100 U/mL (**B**) tyrosinase, respectively. Roughness: 28.7 nm (**A**), 12.8 nm (**B**). Film thickness: 63.3 ± 5 nm (**A**), 58 ± 4 nm (**B**).

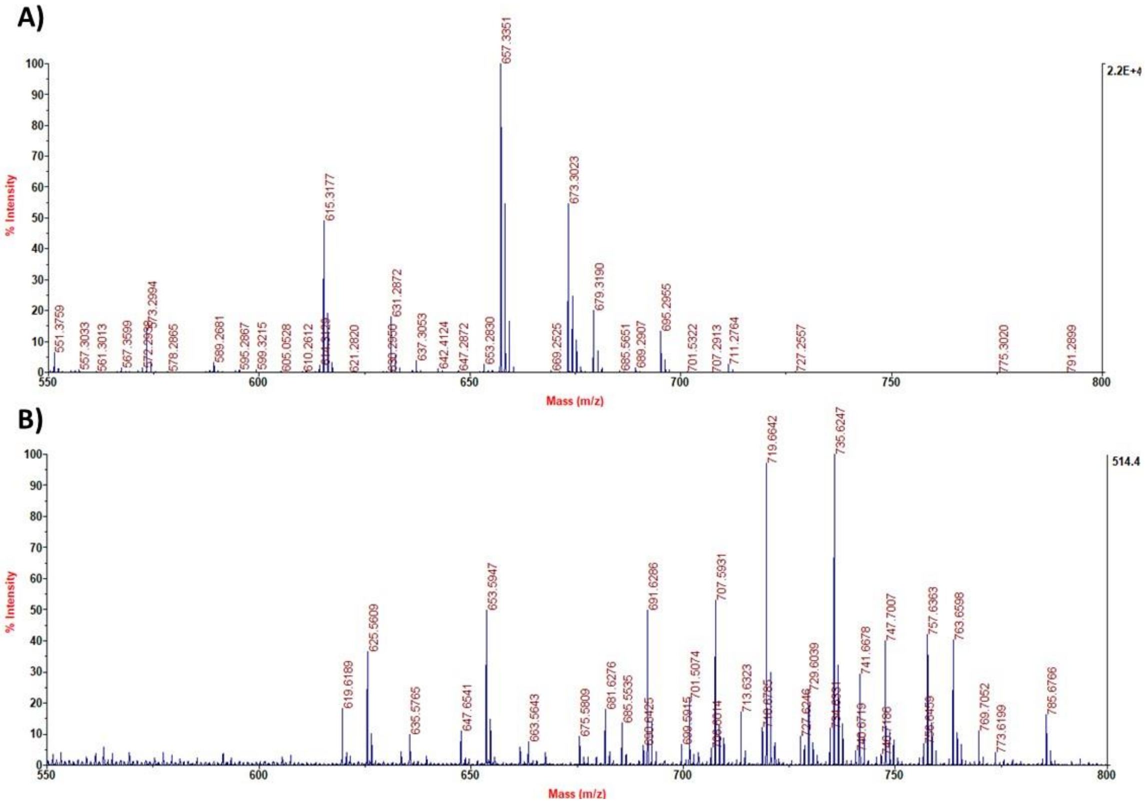

Figure S4. Segmental spectra of MALDI-ToF (*m*/*z*: 550–800 Da) characterizations of (**A**) tyramine film in carbonate buffer at pH = 6.8 with 20 U/mL of tyrosinase and (**B**) PDA film obtained under the same conditions.

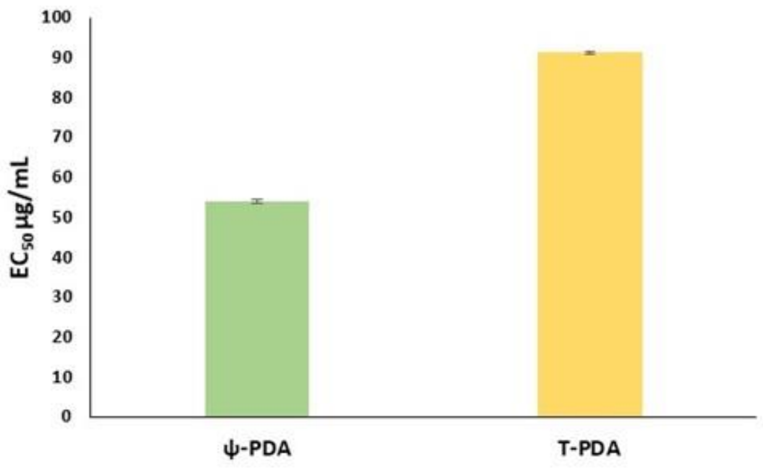

Figure S5. EC_{50} values obtained from the DPPH assay of the samples obtained by oxidation of tyramine and dopamine in the presence of 20 U/mL of tyrosinase (2 mg/mL). The average values ± SD obtained from at least three separate experiments are reported.

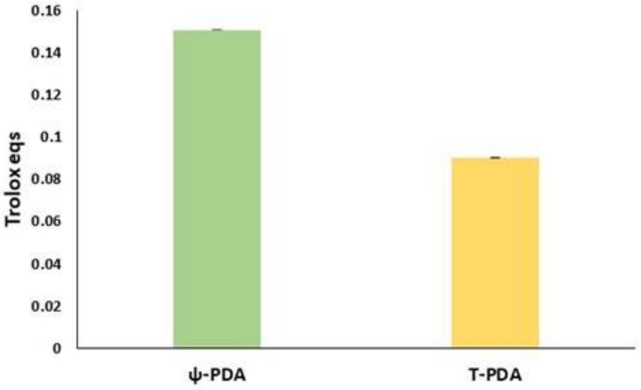

Figure S6. Trolox equivalents determined in the FRAP assay of the samples obtained by oxidation of tyramine and dopamine in the presence of 20 U/mL of tyrosinase (2 mg/mL). The average values ± SD obtained from at least three separate experiments are reported.

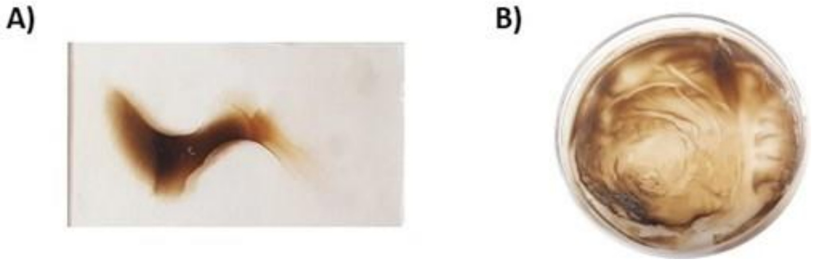

Figure S7. Calcium alginate hydrogel films on glass (**A**) or polystyrene (**B**) loaded with an 1% aqueous solution of tyrosinase and dipped into tyramine solution (1 mM) for 2 h.
